# Genomic Characteristion of Opportunistic Pathogen *Kluyvera* Reveals a Novel CTX-M Subgroup

**DOI:** 10.3390/microorganisms11122836

**Published:** 2023-11-22

**Authors:** Keyi Yu, Zhenzhou Huang, Ruiting Lan, J. Glenn Morris, Yue Xiao, Songzhe Fu, He Gao, Xuemei Bai, Kun Li, Duochun Wang

**Affiliations:** 1 National Institute for Communicable Disease Control and Prevention, Chinese Center for Disease Control and Prevention (China CDC), State Key Laboratory of Infectious Disease Prevention and Control, Beijing 102206, China; yky0414@163.com (K.Y.); xiaoyue@icdc.cn (Y.X.); gaohe@icdc.cn (H.G.); baixuemei@icdc.cn (X.B.); lukeleego@hotmail.com (K.L.); 2 Hangzhou Center for Disease Control and Prevention, Hangzhou 310021, China; ronnie_0414@163.com; 3 School of Biotechnology and Biomolecular Sciences, University of New South Wales, Sydney, NSW 2052, Australia; r.lan@unsw.edu.au; 4 Emerging Pathogens Institute, University of Florida, Gainesville, FL 32610, USA; jgmorris@epi.ufl.edu; 5 College of Marine Science and Environment, Dalian Ocean University, Dalian 116023, China; fusongzhe@dlou.edu.cn

**Keywords:** *Kluyvera*, drug resistance, ESBLs, *bla*_CTX-Ms_

## Abstract

A rising incidence of clinical infections has been caused by *Kluyvera*, a significant opportunistic pathogen. Meanwhile, *Kluyvera* acts as an important reservoir of *bla*_CTX-Ms_, which are the dominant genes of class A extended-spectrum β-lactamases (ESBLs). In this work, 60 strains of *Kluyvera* were subjected to phylogenetic relationship reconstruction, antimicrobial susceptibility testing, and antibiotic resistance genes prediction. All mature *bla*_CTX-Ms_ were gathered to perform subgroup reclassification. The findings demonstrate that *Kluyvera* has a large gene pool with significant genetic flexibility. Notably, 25% of strains showed simultaneous detection of ESBLs and carbapenem resistance genes. The genotypes of fourteen novel *bla*_CTX-Ms_ were identified. A new subgroup classification approach for *bla*_CTX-Ms_ was defined by using 20 amino acid site variants, which could split *bla*_CTX-Ms_ into 10 subgroups. The results of the subgroup division were consistent with the phylogenetic clustering. More significantly, we proposed a novel *bla*_CTX-M_ subgroup, KLUS, that is chromosomally encoded in *K. sichuanensis* and the new species put forward in this study, showing amino acid differences from the currently known sequences. Cloning and transformation tests demonstrated that the recipient bacteria had a robust phenotype of cefotaxime resistance. Closely related *Kluyvera* species had *bla*_CTX-Ms_ in the same subgroup. Our research lays the groundwork for a deeper comprehension of *Kluyvera* and emphasizes how important a *bla*_CTX-M_ reservoir it is. We provide an update on *bla*_CTX-M_ subgroups reclassification from the aspects of phylogenetic relationship, amino acid differences, and the new subgroup KLUS, which needs to be strengthen monitored due to its strong resistance phenotype to cefotaxime.

## 1. Introduction

In recent years, the widespread and inappropriate use of antibiotics has contributed to the spread of antibiotic resistance in pathogens, leading to problems in dealing clinically with antibiotic-resistant infections [1]. Cross-species horizontal transmission of antibiotic resistance genes (ARGs) and antibiotic selective pressures in survival settings facilitate the evolution of bacterial resistance, resulting in greater environmental adaptability [2]. Therefore, the reservoir of ARGs, particularly the association between ARG progenitors and recipients, and the mechanisms of resistance genes transfering from horizontal to vertical are all important in the acquisition of antibiotic resistance.

*Kluyvera* spp. are opportunistic pathogens and can be a part of the normal flora of the human digestive tract [3]. They can be isolated from various clinical and environmental samples including freshwater, seawater, sewage, soil, and the rhizosphere [3]. Since the genus was redefined in 1981 [3], case reports of clinical infections occurring in various host conditions have been reported, shedding light on the importance of this organism as a cause of human disease [3]. While infections have usually involved the gastrointestinal tract, urinary tract, or soft tissues, bacteremia and serious multiple organ system infections have been reported [4,5]. In recent years, more and more *Kluyvera* infection cases have been reported, even fatal cases, highlighting the role of this genus as an emerging opportunistic pathogen and its underestimated pathogenicity [6]. At the time of writing, the genus of *Kluyvera* contains five recognized species (*K. ascorbata*, *K. cryocrescens*, *K. georgiana*, *K. intermedia*, and *K. sichuanensis*) and five genomospecies [7].

Intriguingly, *Kluyvera* is an important reservoir and communicator of *bla*_CTX-Ms_ [8]. *bla*_CTX-Ms_ are currently the dominant resistance genes of class A extended-spectrum β-lactamases (ESBLs), and a large number of *bla*_CTX-M_ variants have emerged and become prevalent, representing an important public health concern [9]. Phylogenetic analysis has shown that *bla*_CTX-Ms_ do not originate from mutations in plasmid-mediated enzymes, but instead are activated through the integration of the chromosomal *bla* gene from *Kluyvera* spp. into mobile genetic elements [8]. Different chromosomal *bla*_CTX-M_-related genes have been identified in different *Kluyvera* species [7].

This study lays the focus on *Kluyvera* taxonomy and the variety of *bla*_CTX-Ms_. We collected all available genomes combined with four newly sequenced strains of *Kluyvera* to determine current species status and perform phylogenetic relationship reconstruction. Meanwhile, using phylogenomics and antibiotic resistance gene screening, we characterized many new *bla*_CTX-M_ genotypes and identified a new CTX-M subgroup, KLUS, which were previously unacknowledged. Our study provides the basis for a better understanding of *Kluyvera* and highlights its role as an abundant reservoir of CTX-Ms.

## 2. Materials and Methods

### 2.1. Genome Sequencing and Curation of Available Kluyvera Genomes

A total of 77 genomes were collected from the NCBI FTP server (ftp.ncbi.nih.gov, accessed on 14 January 2023) (contigs < 200). Four newly sequenced strains isolated from China (deposited in the Centre for Human Pathogenic Culture Collection, China CDC), which were initially identified as *Kluyvera* sp., were included. Detailed information on strains is listed in Table S1. The genomic DNA of four isolated strains was extracted using the Wizard Genomic DNA Extraction Kit (Promega, Madison, WI, USA), following the manufacturer’s instructions. The extracted DNA was then subjected to 250-bp paired-end whole genome sequencing with 150× coverage using the HiSeq sequencer (Illumina HiSeq2000, Illumina, San Diego, CA, USA). Reads were assembled de novo into contigs using SPAdes v3.13.0 [10].

### 2.2. Phylogenomic Analysis

Average nucleotide identity (ANI) calculations [11] and the genome-to-genome blast distance phylogeny (GBDP) algorithm which replicates the DNA–DNA hybridization digitally for species delineation [12] were used for phylogenomic analysis. Minimal cutoff points of 70% for digital DNA–DNA hybridization (dDDH) and 95% for ANI values were considered to represent species delineation. Intergenomic distances were calculated using the genome-to-genome distance calculator web service (GGDC: http://ggdc.dsmz.de/, accessed on 6 February 2023) [12] and were then transformed into a matrix and used to build a neighbor-joining (NJ) phylogenomic tree through FastMe (v2.0) [13]. The online TYGS platform (https://tygs.dsmz.de/user_requests/new, accessed on 14 February 2023) was used to assess strain identification at the genus or species level [14]. Clustering of intergenomic distances was examined using OPTSIL (v1.5) software [15], which creates a non-hierarchical clustering using distance threshold T and specific F value. The distance threshold (T) values were from 0 to 0.3, using a step size of 0.0005. F values ranging from 0 to 1 indicated the fraction of links required for cluster fusion. F values of 0.5 represented average-linkage clustering.

### 2.3. Evolutionary Analysis

Prokka (v1.12) [16] was used to annotate genomes and produces *.gff output files for each strain. Roary pan-genome pipeline [17] was used to perform analysis. Coding sequence prediction was carried out using Prodigal (v2.6.3) [18]. Then, the core genes were extracted using CD-HIT (v4.6.6) to build a non-redundant homologous gene set [19]. Next, BLAST+ was used to search the homologous genes in the non-redundant homologous gene set, with ≥90% sequence identity and ≥60% length coverage. The core genes were then aligned, and Gubbins (http://github.com/sanger-pathogens/gubbins, accessed on 10 March 2023) was used as a recombination-removal tool to reorganize the core genome. PhyML (v3.1) [20] was used to construct the phylogenetic trees using the maximum likelihood method based on all these core SNPs (bootstrap replications, 1000). Population structure was defined using FastBaps (https://github.com/gtonkinhill/fastbaps, accessed on 26 April 2023) through a fast hierarchical Bayesian analysis [21]. Snippy software (v4.3.6) was used to extract core gene SNPs to build a maximum likelihood phylogenetic tree based on core gene SNP alignment and concatenation.

### 2.4. Identification of Antibiotic Resistance Genes and In Vitro Antibiotic Susceptibility Test

Antibiotic resistance genes were predicted using the Comprehensive Antibiotic Research Database (CARD) (http://arpcard.mcmaster.ca, accessed on 13 May 2023), with an E-value of 1 × 10^−5^, sequence identity of ≥80%, and length coverage of ≥80%. Currently known sequences of *bla*_CTX-Ms_ in the Beta-Lactamase DataBase (http://bldb.eu/, accessed on 20 May 2023) were collected to perform phylogenetic analysis. Four strains collected in this study (CHPC 1.251, CHPC 1.254, CHPC 1.2972, and CHPC 1.982) were tested for antimicrobial susceptibility using an antimicrobial susceptibility testing (AST) panel for aerobic Gram-negative bacilli (Shanghai Fosun Long March Medical Science Co., Ltd., Shanghai, China) with the microbroth dilution method. Twenty-one antibiotics were used in testing (amikacin, ampicillin, ampicillin-sulbactam, azithromycin, aztreonam, cefotaxime, cefoxitin, ceftazidime, chloramphenicol, ciprofloxacin, colistin, ertapenem, imipenem, meropenem, nalidixic acid, nitrofurantoin, streptomycin, tetracycline, tigecycline, trimethoprim-sulfamethoxazole, and ceftazidime-avibactam). *Escherichia coli* ATCC 25922 was used as the quality control strain.

### 2.5. CTX-Ms Cloning and Phenotypic Verification

PCR was performed for four *bla*_CTX-M_ variants (CHPC 1.251, CHPC 1.254, CHPC 1.982, and CHPC 1.2972) with self-designed primers (Table S2), and amplification products were cloned into vector pSRKGm. The *E. coli* strain DH5α served as the cloning host. Luria Bertani (LB) broth supplemented with appropriate antibiotics was used to screen *bla*_CTX-M_ variant-containing clones and determine the level of cefotaxime resistance. The phenotype of bacterial insusceptibility to cefotaxime was assessed through a Mueller–Hinton broth microdilution and interpreted as recommended by the 2021 guidelines of the Clinical & Laboratory Standards Institute (CLSI) [22].

### 2.6. Data Availability

The genome sequences of four *Kluyvera* strains sequenced in this study have been deposited at GenBank/DDBJ/ENA under the BioProject ID no. PRJNA899585 and the accession numbers JAPKIA000000000 (*Kluyvera* genomosp. 2 CHPC 1.982), JAPKIB000000000 (*Kluyvera* sp. CHPC 1.2972), JAPKIC000000000 (*Kluyvera* sp. CHPC 1.251), and JAPPVH000000000 (*Kluyvera* sp. CHPC 1.254).

## 3. Results

### 3.1. Genomic Characteristics and Pan-Genome Analysis Highlights Genetic Divergence

**Figure 1:**
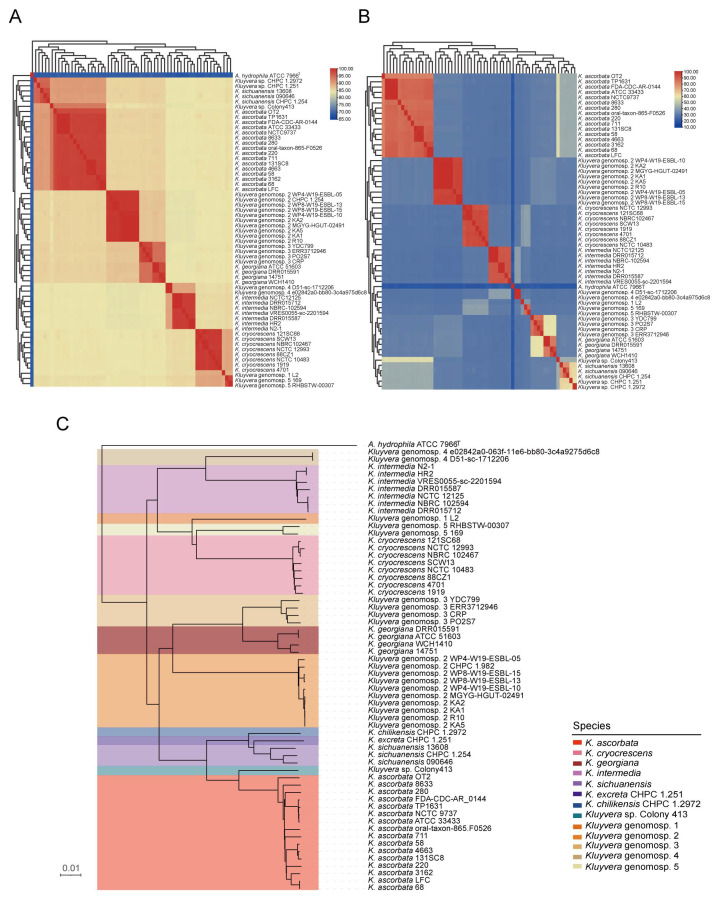
ANI-, dDDH- and GBDP-based phylogenetic analyses of *Kluyvera* strains. *Escherichia coli* MG1565 served as the outgroup. (**A**) Species-level clusters with an ANI threshold at 95%. *Aeromonas hydrophila* ATCC 7966^T^ served as an outgroup. (**B**) Species-level clusters with a dDDH threshold at 70%. *A. hydrophila* ATCC 7966^T^ served as an outgroup. (**C**) GBDP-based phylogeny of *Kluyvera* genomes. Clustering analysis was based on GBDP intergenomic distances with threshold T = 0.036, MRI = 0, and single-lineage clustering (F = 0). Different species were represented by different colors.

Based on the ANI and dDDH analysis (Figure 1A,B) using the 95% and 70% cutoff values, some strains were misclassified, resulting in 60 *Kluyvera* strains that were eventually identified as members of the genus *Kluyvera* (Figure 1A,B) (Table S1). The strains CHPC 1.251, isolated from human feces, and CHPC 1.2972, isolated from the egg surface, achieved dDDH below 70% (63.7% and 61.9%, respectively) and an ANI below 95–96% (94.88% and 94.56%, respectively) compared to their closely related species *K. sichuanensis* strain 090646^T^, suggesting that these two strains represent two novel species of the genus *Kluyvera*. A phylogenomic tree was built based on the intergenomic distances (Figure 1C) using the GBDP algorithm [12]. At the species level, based on the distance threshold T = 0.036 which equals 70% of digital DNA–DNA hybridization, there were 13 species clusters (i.e., MRI = 0) when using single-lineage clustering (F = 0), further supporting CHPC 1.251 and CHPC 1.2972 as new species from a genomic perspective. Combined with the phenotypic characteristic differences (Table S3), the names *K. excreta* and *K. chilikensis* were proposed (*K. excreta* CHPC 1.251^T^ = GDMCC 1.3297^T^; *K. chilikensis* CHPC 1.2972^T^ = GDMCC 1.3298^T^).

**Figure 2:**
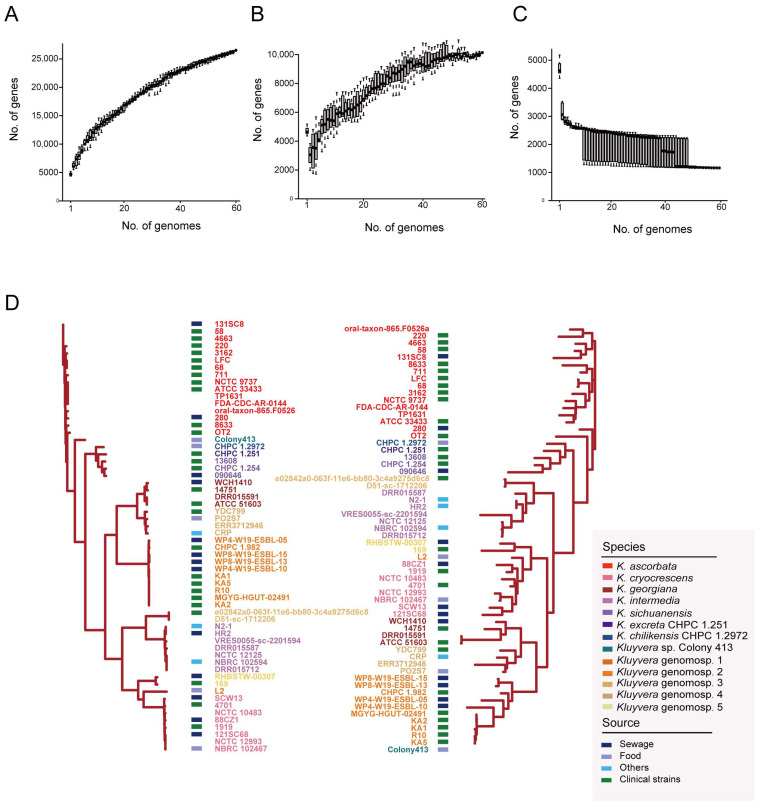
Pan-genome analysis of *Kluyvera* strains. (**A**) The dilution curve of pan genes. (**B**) The dilution curve of unique genes. (**C**) The dilution curve of core genes. (**D**) Phylogenetic tree of core genome sequences (left) and accessory genes (right) using the maximum likelihood method. Different species were represented by different colored words. The sources of isolates were marked with different colored squares.

A total of 26,512 pan genes and 1164 core genes were identified among the 60 *Kluyvera* strains. According to the dilution curves, the pan-genome curve showed a constantly increasing slope (Figure 2A), indicating that the genome sequences included in this study failed to represent the genetic diversity of the whole population. The strain-specific unique gene curve (Figure 2B) showed a similar pattern, which means that the number of genomes currently being studied were not enough to cover the shared diversity. Conversely, the core-genome curve with a steadily decreasing trend (Figure 2C) showed that the strains in this study achieved a representation of core diversity.

The 79,783 core gene SNPs were used to analyze the genetic relationships of the *Kluyvera* strains (Figure 2D). The clustering patterns were almost identical to those based on whole-genome sequences. The clade of *K. ascorbata* showed deeper evolutionary roots. However, in terms of the phylogenetic tree topology, certain clusters of the accessory genome-based phylogenetic tree were different from those of the core genome-based phylogenetic tree (Figure 2D). The branch lengths of the accessory genome-based phylogenetic tree were longer than those of the core genome-based phylogenetic tree, representing longer genetic distances. In the accessory genome-based phylogenetic tree, strains of the same species and the same niches were clustered together, suggesting similar accessory gene compositions.

### 3.2. Analysis of Antibiotic Resistance Genes (ARGs) in Kluyvera

**Figure 3:**
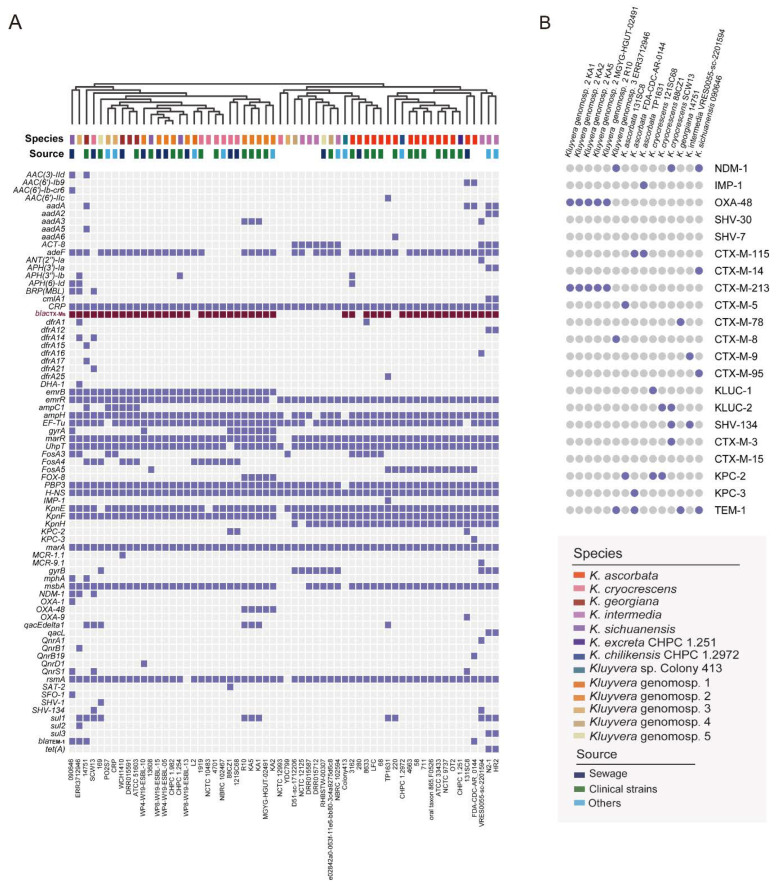
Antibiotic resistance genes (ARG) landscape in *Kluyvera*. (**A**) Distributions of ARGs in *Kluyvera* strains. (**B**) Coexistence patterns of several ESBLs, *bla*_NDM_, *bla*_KPC_, and *bla*_IMP_.

We undertook a combined genomic and phenotypic analysis of resistance genes. A total of 94 ARGs were identified by searching against the CARD (Figure 3A). Two antibiotic resistance ontologies (AROs) (H-NS, CRP), with the common resistance mechanism of a resistance-nodulation-cell division (RND) antibiotic efflux pump, were identified in the core genome.

The co-existence of multiple antibiotic resistance genes occurred more frequently in strains isolated from sewage than in strains from other origins (Figure 3A). Many strains were potentially multidrug-resistant, harboring three or more classes of antimicrobial resistance genes. Several ESBL genes were detected (Figure 3B). Notably, ESBLs and carbapenem resistance genes (*bla*_KPC_, *bla*_NDM_, *bla*_IMP_, and *bla*_OXA-48_) were synchronously detected in 15 strains (Figure 3B). The antimicrobial susceptibility of four strains is shown in Table S4. All strains were resistant to azithromycin. The strains CHPC 1.251 and CHPC 1.2972 were also resistant to ampicillin and cefoxitin.

### 3.3. Analysis of Native bla_CTX-Ms_ in Kluyvera

**Figure 4:**
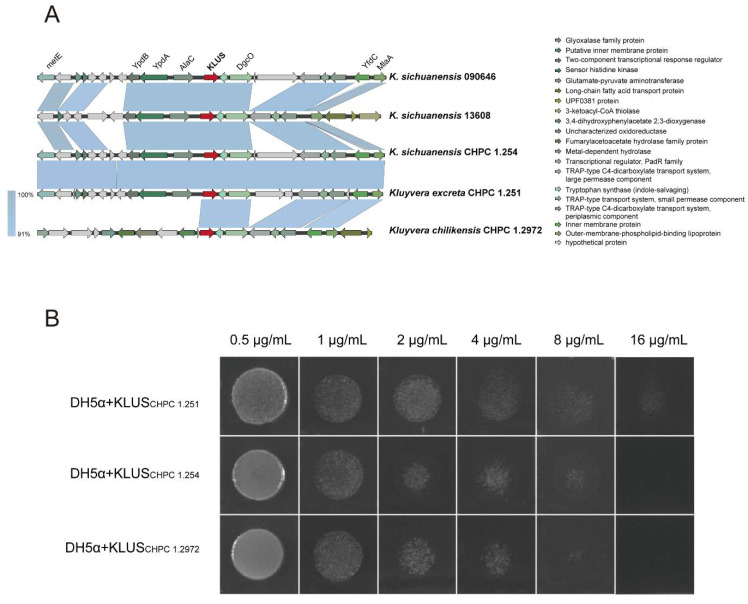
(**A**) Comparative analysis of genetic context in the newly discovered CTX-M subgroup KLUS. (**B**) The phenotype of cefotaxime resistance verified by cloning the KLUS genes of the strains CHPC 1.251, CHPC 1.254, and CHPC 1.2972 into DH5α.

The *bla*_CTX-Ms_ were found in 44 strains. Pairwise amino acid differences were calculated between the potential new *bla*_CTX-Ms_ and the closely related known *bla*_CTX-Ms_. Fourteen new variants were characterized (Table 1). The genetic backgrounds of *bla*_CTX-Ms_ identified in *K. sichuanensis* and two novel species were also explored. The results showed that *bla*_CTX-Ms_ were chromosomally encoded, with no surrounding mobile genetic elements (Figure 4A). Next, we cloned the *bla*_CTX-Ms_ encoded in the chromosomes of CHPC 1.251, CHPC 1.254, and CHPC 1.2972 into *E. coli* DH5α, and the recipient *E. coli* showed a strong cefotaxime-resistant phenotype (MIC are 16 μg/mL, 8 μg/mL, and 8 μg/mL, respectively) (Figure 4B).

### 3.4. Reclassification of bla_CTX-Ms_

**Figure 5:**
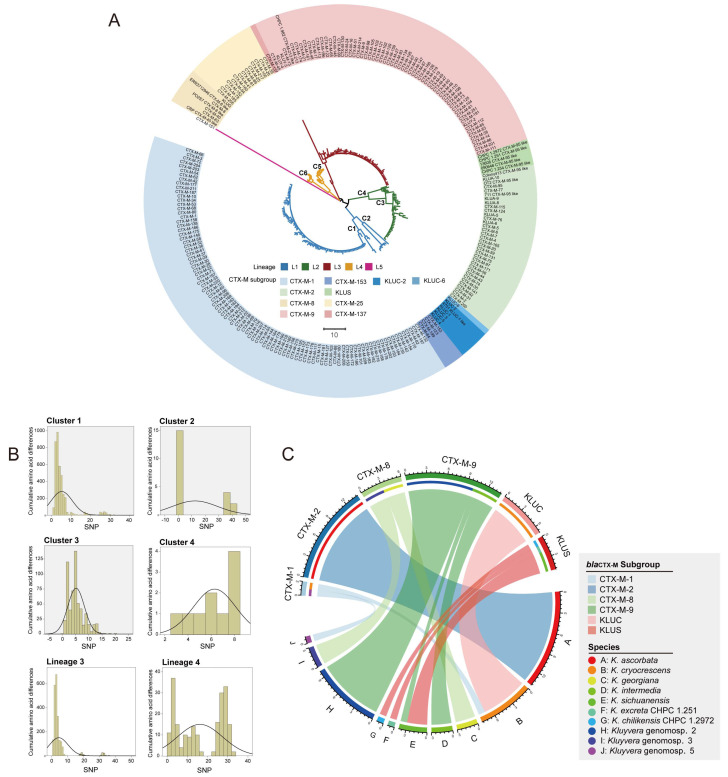
(**A**) Phylogenetic analysis of the amino acid sequences of all currently available *bla*_CTX-Ms_ and the newly discovered *bla*_CTX-Ms_ in this study using the maximum likelihood method. (**B**) Comparative analysis of the genetic content in the newly discovered CTX-M subgroup KLUS. (**C**) Each species contributes to the recruitment and dissemination of CTX-M resistance genes.

To provide insights into the evolutionary relationships of CTX-Ms, a phylogenetic tree based on mature *bla*_CTX-Ms_ and new genotypes was built, and five lineages were identified (Figure 5A). Based on the past subgroup classification criteria, the recognized subgroups (CTX-M-1, CTX-M-2, CTX-M-8, CTX-M-9, CTX-M-25, and KLUC) were scattered into different lineages, forming independent clusters. Lineage 1 contained the CTX-M-1 subgroup and KLUC subgroup, lineage 3 contained the CTX-M-9 subgroup, and lineage 4 contained the CTX-M-8 and CTX-M-25 subgroups. It is worth noting that lineage 2 was divided into two distinct clusters, which were *bla*_CTX-M-2_ and *bla*_CTX-Ms_ identified in *K. sichuanensis* and the two novel species.

We calculated the amino acid differences of clusters (Table 2) and also performed frequency distribution statistics for paired SNPs of the *bla*_CTX-M_ genotypes in the same cluster and same lineage (Figure 5B) based on the amino acid sequences. In previous studies, researchers have typically used amino acid sequences with a similarity greater than 95% as the basis for classifying *bla*_CTX-M_ subgroups [23,24]. By calculating the amino acid sequence similarity within the subgroups, we learned that only the similarity of the sequences within the subgroups of CTX-M-1 and CTX-M-8 were 98.23–99.66% and 97.25–99.66%, respectively, and the sequence similarity of *bla*_CTX-Ms_ within the rest of the subgroups could no longer reach more than 95% (Table 2). The lowest similarity of sequences within the CTX-M-9 subgroup was 84.88%, suggesting that new subgrouping rules need to be defined. We found that CTX-M genotypes clustered in the same branch all differed by less than 20 amino acid loci. In light of the monophyletic clades and amino acid differences, we reclassified the CTX-M subgroups into 10 based on 20 (6.87%, 20/291) amino acid site variants. Liu et al. proposed a novel species, *Kluyvera sichuanensis,* in 2020, which harbors an intrinsic chromosomal *bla*_CTX-M_ sharing the highest amino acid identity (90.72%, 264/291) with CTX-M-95 [25]. Combined with the strain *K. sichuanensis* 13608 [7] and the self-isolated strains *K. sichuanensis* 1.254, *K. excreta* CHPC 1.251, and *K. chilikensis* CHPC 1.2972, the chromosomes of the above five strains all contained *bla*_CTX-Ms_ encoding a CTX-M enzyme with amino acid differences from the currently known *bla*_CTX-Ms_, so we named it the novel family KLUS. Thus, the newly defined *bla*_CTX-M_ subgroups are CTX-M-1, CTX-M-2, CTX-M-8, CTX-M-9, CTX-M-25, CTX-M-153, KLUC-2, KLUC-6, KLUS, and CTX-M-137. This new delineation of CTX-M subgroups is an update of the six subgroups defined in previous studies [24]. Most *bla*_CTX-M_ variants of the CTX-M-1, CTX-M-2, CTX-M-8, and CTX-M-9 subgroups have few amino acids differences. The subgroups of CTX-M-1, CTX-M-2, CTX-M-8, and CTX-M-9 are still the dominant subgroups with a high number of variants. However, on the whole, the amino acid differences among the variants in the above subgroups were small, showing convergent evolution.

### 3.5. Clarification on Each Species’ Contribution to the Recruitment of bla_CTX-Ms_

Chromosomal-derived *bla*_CTX-Ms_ from different *Kluyvera* species were derived from different *bla*_CTX-M_ clusters, based on hierarchical clustering using 20 amino acid loci as a cutoff. Genomic analysis was used to compare *bla*_CTX-Ms_ present in the selected sequences and clarify each species’ contribution to the recruitment and dissemination of *bla*_CTX-M_ resistance genes (Figure 5C). The chord diagram was used to show the distribution. Unlike the *bla*_CTX-M-2_ and *bla*_KLUC_ subgroups, which were only identified in *K. ascorbata* and *K. cryocrescens*, respectively, the remaining *bla*_CTX-Ms_ were distributed in several *Kluyvera* species. The CTX-M-1 subgroup was linked to *Kluyvera* genomospecies-5 and *K. cryocrescens*, while the CTX-M-8 subgroup was linked to *Kluyvera* genomospecies-3 and *K. georgiana*. CTX-M-9 was harbored by *Kluyvera* genomospecies-2, *K. sichuanensis,* and *K. intermedia*. KLUS was mainly identified in *K. sichuanensis*, *K. extract* CHPC 1.251, and *K. chilikensis* CHPC 1.2972.

## 4. Discussion

As the number of infection cases have increased, *Kluyvera* has become a compelling and enigmatic human pathogen, yet its biology, genomics, virulence, and epidemiology remain poorly understood. In this study, we performed a comprehensive analysis of the evolution, virulence, and resistance genes of *Kluyvera*. The pan-genome analysis showed that the high genome plasticity of *Kluyvera* may relate to the diverse ecological niches in which it can be found. The long branch lengths in the accessory gene-based phylogenetic tree represent long genetic distances and indicate that strains of the same species may be affected by survival pressure to acquire more diverse accessory genes. It should be admitted that the number of newly sequenced strains is small, which is a limitation of this study. In the process following, we will continue to collect more strains to enrich the analysis.

In recent years, ESBL-producing micro-organisms have emerged around the world and posed a great threat to public health. ESBLs include TEM, SHV, CTX-M, VEB, and GES enzymes [24,26], in which the CTX-M family harbored the highest number of variants [27]. At present, more than 200 genotypes have been discovered (http://bldb.eu/Enzymes.php) and have been distributed among at least five subgroups (CTX-M-1, CTX-M-2, CTX-M-8, CTX-M-9, and KLUC). The manifestation of *Kluyvera* as an opportunistic pathogen may also be associated with its genome acting as a reservoir for resistance genes (*bla*_CTX-Ms_) encoding ESBL enzymes that confer resistance to first-, second-, third-generation cephalosporins and aztreonam. Previous studies suggest that *bla*_CTX-Ms_ in *Kluyvera* are chromosomally encoded and not recently acquired from other sources [26]. Rather, plasmid-encoded *bla*_CTX-Ms_ in other species are sourced from the *bla*_CTX-Ms_ in *Kluyvera* [9]. In this study, 14 new CTX-M genotypes were discovered, and a reclassification system was established to divide the CTX-Ms into 10 subgroups based on an amino acid difference of less than 20 AAs (identity > 93.13%). Previously, *bla*_CTX-M-2_ was found in *K. ascorbata*, *bla*_KLUC_ was found in *K. cryocrescens,* and the *bla*_CTX-M-8_ subgroup was identified in *K. georgiana* [9,28,29]. In this study, the subgroups CTX-M-2 and KLUC show the specific distribution in different *Kluyvera* species, consistent with what has been described previously. The rest of the *bla*_CTX-Ms_ were found in different *Kluyvera* species. The CTX-M-1 subgroup was linked to *Kluyvera* genomospecies-5 and *K. cryocrescens*, while the CTX-M-8 subgroup was linked to *Kluyvera* genomospecies-3 and *K. georgiana*. The newly discovered and named CTX-M subgroup KLUS in this study was first detected in *K. sichuanensis* 090646 [25]; therefore, we named it KLUS. The genes belonging to KLUS were mainly identified in *K. sichuanensis*, *K. excreta* CHPC 1.251, and *K. chickenensis* CHPC 1.2972. Closely related *Kluyvera* strains have CTX-Ms belonging to the same subgroup. However, CTX-M-9 had a more complex distribution compared to other *bla*_CTX-Ms_ and was harbored by *Kluyvera* genomospecies-2, *K. sichuanensis,* and *K. intermedia*. We speculated that *Kluyvera* strains carrying *bla*_CTX-Ms_ could provide a selective advantage and be more adaptable to natural selection than *Kluyvera* strains without *bla*_CTX-Ms_. The evolution of chromosomally encoded *bla*_CTX-Ms_ in *Kluyvera* spp. may ensure the balance between ecological niches and biological functions. Interestingly, the strain *K. sichuanensis* 090646^T^, carrying *bla*_CTX-Ms_ (one on the chromosome and one on the plasmid) and *bla*_NDM-1_, was isolated from the sink of a hospital in Chengdu, Sichuan Province, China, and showed strong drug resistance (resistant to ampicillin, azithromycin, aztreonam, cefotaxime, imipenem, streptomycin, etc.). We suspected that sewage provides sufficient conditions for resistance selection and imposes a strong selective pressure that drives the evolution and spread of antibiotic resistance.

In this study, we identified new CTX-M genotypes based on amino acid sequence comparisons. A total of 14 new *bla*_CTX-M_ genotypes were discovered. Genotypes of the same subgroup have small differences in amino acid sequences and show convergent evolutionary patterns (Table 2). The members of the other subgroups have an approximately 90% sequence identity. The diversity of *bla*_CTX-Ms_ present in the *Kluyvera* genomes suggests that many *bla*_CTX-Ms_ can potentially spread to other pathogens, which would reduce clinical treatment options. The evolution of antibiotic resistance genes is proceeded by random (mutation and drift) and directional (natural selection) processes. Sometimes, sequential pathways of adaptive variation can occasionally be observed. The changing niches and selective gradients in complex environments make the evolutionary trajectories of antibiotic resistance genes unpredictable. This process of emergence of resistance genes can be accelerated by combinatorial events involving the building up of complex (chimeric) proteins from sequences determining protein domains and also by combinations of pre-existing genes [1], which coincides with the evolutionary pattern of the rapidly expanding CTX-M gene family. The phylogenetic tree of all known *bla*_CTX-Ms_ showed that *bla*_CTX-Ms_ can be divided into five lineages, with the main *bla*_CTX-M_ subgroups scattered in each lineage. Based on the frequency distribution histogram, which was constructed using the number of amino acid site differences in the same subgroup, we redefined *bla*_CTX-Ms_ into 10 subgroups based on amino acid site changes of less than 20 (identity > 93.13%). The CTX-M-1 subgroup had the highest number of variants. Most of them only had one amino acid site difference, indicating a convergent evolutionary trend. The KLUS subgroup was assigned to be a new single subgroup, consisting of CTX-Ms identified in *K. sichuanensis* and two proposed novel species.

The co-existence of two or more genes of β-lactamases in the same strain is a possible way for common bacteria to enhance antibiotic resistance. In our study, each strain was found to carry multiple resistance genes, which suggests that these strains retained the possibility of becoming multidrug resistant. Carbapenems, which can be hydrolyzed by carbapenemase, are generally considered as the most effective option for infections caused by ESBL-producing enterohepatic bacteria [6]. The co-existence of several ESBLs and carbapenem resistance genes (*bla*_KPC_, *bla*_NDM_, *bla*_IMP_, and *bla*_OXA-48_) was found in a quarter of the strains and was more common in strains isolated from sewage. These strains have the potential to develop into extensive drug-resistant or even pandrug-resistant strains. Permanent selective pressure existing in complex environments like sewage would appear to drive diversification of the resistance mechanisms and reinforce horizontal transfer and acquisition of resistance genes. Due to the limited number of strains in this study, the drug resistance phenotypes should be further verified. Despite harboring ESBLs, the two *Kluyvera* strains of novel species identified in this study were only resistant to ampicillin, azithromycin, and cefoxitin. Interestingly, they did not confer resistance to cefotaxime despite carrying CTX-Ms. It has been demonstrated that naturally chromosomally encoded *bla*_CTX-Ms_ in the ancestral species are not expressed, resulting in the narrow spectrum of resistance [15]. This phenotype has been observed in many genes of ancestral species [30]. Over time, this is how they might have been selected. In most cases, an insertion sequence (IS) introduces a promoter that enhances resistance levels. This genetic structure would then be favored and likely promote the mobilization of these genes into other genetic structures, such as plasmids [31]. As an important reservoir of resistance genes and a potential multidrug-resistant opportunistic pathogen, *Kluyvera* represents a potential threat to human health.

## 5. Conclusions

Our research lays the groundwork for a deeper comprehension of *Kluyvera* and emphasizes how important a *bla*_CTX-M_ reservoir it is. We provide an update on *bla*_CTX-M_ subgroup reclassification from the aspects of phylogenetic relationship, amino acid differences, and the new subgroup KLUS, which needs to be strengthen monitored, due to its strong resistance phenotype to cefotaxime.

## Figures and Tables

**Table 1:** Sequence similarity between 14 new *bla*_CTX-Ms_ and closely related known *bla*_CTX-Ms_.

Closely Related CTX-Ms	Strains	Identity	SNP
CTX-M-95	*K. ascorbata* 711	99.31	2
	*K. ascorbata* oral-taxon-865.F0526	99.31	2
	*K. sichuanensis* 090646	91.03	27
	*K. sichuanensis* 13608	91.03	27
	*K. excreta* CHPC 1.251	90.34	29
	*K. sichuanensis* CHPC 1.254	90.34	29
	*K. chilikensis* CHPC 1.2972	90.69	28
	*Kluyvera* sp. Colony413	96.56	10
CTX-M-115	*K. ascorbata* OT2	99.31	5
CTX-M-8	*Kluyvera* genomosp. 3 ERR3712946	98.97	3
	*Kluyvera* genomosp. 3 PO2S7	99.31	2
CTX-M-40	*Kluyvera* genomosp. 3 CRP	99.56	1
CTX-M-213	*Kluyvera* genomosp. 2 CHPC 1.982	99.31	2
KLUC-1	*K. cryocrescens* 4701	99.66	1

**Table 2:** The amino acid differences in different lineages.

Lineage	Cluster	Subgroup	Amino Acid Differences	AA%
L1	C1	CTX-M-1	Ranged from 1 to 32	0.34–1.77%
	C2	KLUC	Ranged from 1 to 39	0.34–13.4%
L2	C3	CTX-M-2	Ranged from 1 to 20	0.34–6.87%
	C4	New CTX-M subgroup identified in *K. sichuanensis* and two novel species	Ranged from 3 to 8	1.03–2.75%
L3	-	CTX-M-9	Ranged from 1 to 44	0.34–15.12%
L4	C5	CTX-M-8	Ranged from 1 to 8	0.34–2.75%
	C6	CTX-M-25	Ranged from 1 to 16	0.34–5.50%
L5	-	CTX-M-151	-	-

## Data Availability

The genome sequences of four *Kluyvera* strains sequenced in this study have been deposited at GenBank/DDBJ/ENA under the BioProject ID no. PRJNA899585 and the accession numbers JAPKIA000000000 (*Kluyvera* genomosp. 2 CHPC 1.982), JAPKIB000000000 (*Kluyvera* sp. CHPC 1.2972), JAPKIC000000000 (*Kluyvera* sp. CHPC 1.251), and JAPPVH000000000 (*Kluyvera* sp. CHPC 1.254).

## Supplementary Materials

The following supporting information can be downloaded at: https://www.mdpi.com/article/10.3390/microorganisms11122836/s1. Table S1: Detailed information on the strains used in this study; Table S2: Primer sequence information used in this study; Table S3: Phenotypic characteristics of *K. sichuanensis* 090646^T^, *K. excreta* CHPC 1.251^T^ and *K. chilikensis* CHPC 1.2972^T^; Table S4: The results of antibiotic susceptibility testing.^.^
