# Supplementary material for: Genomic Characteristion of Opportunistic Pathogen Kluyvera Reveals a Novel CTX-M Subgroup
Source: Microorganisms. 2023 Nov 22;11(12):2836. doi: 10.3390/microorganisms11122836 (PMC10745516; doi:10.3390/microorganisms11122836)
Supplement: Supplementary file 1 [file microorganisms-11-02836-s001.zip › microorganisms-2678385-supplementary.pdf]

Genomic Characteristion of Opportunistic Pathogen *Kluyvera* Reveals a Novel CTX-M Subgroup

Keyi Yu [1], Zhenzhou Huang [2], Ruiting Lan [3], J. Glenn Morris, Jr. [4], Yue Xiao [1], Songzhe Fu [5], He Gao [1], Xuemei Bai [1], Kun Li [1] and Duochun Wang [1,*]

[1] National Institute for Communicable Disease Control and Prevention, Chinese Center for Disease Control and Prevention (China CDC), State Key Laboratory of Infectious Disease Prevention and Control, Beijing 102206, China; yky0414@163.com (K.Y.); xiaoyue@icdc.cn (Y.X.); gaohe@icdc.cn (H.G.); baixuemei@icdc.cn (X.B.); lukeleego@hotmail.com (K.L.)

[2] Hangzhou Center for Disease Control and Prevention, Hangzhou 310021, China; ronnie_0414@163.com

[3] School of Biotechnology and Biomolecular Sciences, University of New South Wales, Sydney, NSW 2052, Australia; r.lan@unsw.edu.au

[4] Emerging Pathogens Institute, University of Florida, Gainesville, FL 32610, USA; jgmorris@epi.ufl.edu

[5] College of Marine Science and Environment, Dalian Ocean University, Dalian 116023, China; fusongzhe@dlou.edu.cn

* Correspondence: wangduochun@icdc.cn

Table S1 The detail information of strains used in this study

No.	Strain	Sequence size (Mb)	GC content (%)	BioSample	geographic location	host	isolation time	isolation source	Species labelled (Originally identified)	Precise species
1	220	5.1	54.2	SAMN16845431	Argentina: Buenos Aires	Homo sapiens	1998	peritoneal fluid	*Kluyvera ascorbata*	*Kluyvera ascorbata*
2	280	4.90	54.3	SAMN16845432	Japan: Tokio	environment	1956	Sewage	*Kluyvera ascorbata*	*Kluyvera ascorbata*
3	3162	4.98	54.3	SAMN16845433	USA: SENTRY	Homo sapiens	-	sepsis	*Kluyvera ascorbata*	*Kluyvera ascorbata*
4	4663	4.88	54.2	SAMN16845434	USA: Charlottesville, VA	Homo sapiens	2004	sepsis	*Kluyvera ascorbata*	*Kluyvera ascorbata*
5	58	5.02	54.1	SAMN16845429	Argentina: Buenos Aires	Homo sapiens	2014	bile from acute cholecystitis	*Kluyvera ascorbata*	*Kluyvera ascorbata*
6	68	4.91	54.3	SAMN16845430	Argentina: Buenos Aires	Homo sapiens	1995	sputum	*Kluyvera ascorbata*	*Kluyvera ascorbata*
7	711	4.73	54.4	SAMN16845443	USA: Burlington, VA	Homo sapiens	1999	skin infection and soft tissues, abscess	*Kluyvera ascorbata*	*Kluyvera ascorbata*
8	8633	5.06	54.2	SAMN16845435	USA: Burlington, MA	Homo sapiens	1999	sepsis	*Kluyvera ascorbata*	*Kluyvera ascorbata*
9	ATCC 33433	4.93	54.3	SAMN02743264	USA:North Carolina	Homo sapiens	-	human sputum	*Kluyvera ascorbata*	*Kluyvera ascorbata*
10	FDA-CDC-AR_0144	4.98	54.1	SAMN04014985	-	-	-	-	*Kluyvera ascorbata*	*Kluyvera ascorbata*

11	LFC	4.92	54.2	SAMN16845437	Argentina: Buenos Aires	Homo sapiens	2014	lower urinary tract infection	*Kluyvera ascorbata*	*Kluyvera ascorbata*
12	NCTC9737	5.13	54.1	SAMEA103980416	Uruguay: Montevideo	Homo sapiens	-	urine	*Kluyvera ascorbata*	*Kluyvera ascorbata*
13	oral taxon 865. F0526	5.25	54.2	SAMN00691209	-	-	-	-	*Kluyvera ascorbata*	*Kluyvera ascorbata*
14	OT2	5.37	53.9	SAMN10265530	Brazil:Porto Alegre	Homo sapiens	2016	abdominal collection	*Kluyvera ascorbata*	*Kluyvera ascorbata*
15	TP1631	5.37	54.0	SAMD00204448	Japan	-	2017	-	*Kluyvera ascorbata*	*Kluyvera ascorbata*
16	131SC8	5.47	53.7	SAMEA104032393	Spain	environment	2013	Sewage	*Kluyvera ascorbata*	*Kluyvera ascorbata*
17	121SC68	5.18	53.7	SAMEA104032383	Spain	environment	2012	Sewage	*Kluyvera cryocrescens*	*Kluyvera cryocrescens*
18	1919	4.97	53.7	SAMN16845439	USA: San Francisco, CA	Homo sapiens	2002	sputum (lower respiratory tract infection)	*Kluyvera cryocrescens*	*Kluyvera cryocrescens*
19	4701	4.80	54.0	SAMN16845440	USA: Milwaukee, WI	Homo sapiens	2002	sepsis from intravenous catheter	*Kluyvera cryocrescens*	*Kluyvera cryocrescens*
20	88CZ1	5.23	53.6	SAMEA104032394	Spain	environment	2011	Sewage	*Kluyvera cryocrescens*	*Kluyvera cryocrescens*
21	NBRC 102467	5.04	53.9	SAMD00046725	-	food	2006	gulf	*Kluyvera cryocrescens*	*Kluyvera cryocrescens*
22	NCTC10483	4.82	54.0	SAMEA103924398	Japan: Tokyo	-	-	-	*Kluyvera cryocrescens*	*Kluyvera cryocrescens*
23	NCTC12993	5.29	53.8	SAMEA2580319	-	-	-	-	*Kluyvera cryocrescens*	*Kluyvera cryocrescens*
24	SCW13	5.44	53.4	SAMN12268149	China:Sichuan	environment	2019	hospital sewage	*Kluyvera cryocrescens*	*Kluyvera cryocrescens*

25	14751	5.00	55.1	SAMN16845442	USA: Louisville, KY	Homo sapiens	2002	sepsis	*Kluyvera georgiana*	*Kluyvera georgiana*
26	ATCC 51603	5.07	54.5	SAMN04875430	-	Homo sapiens	-	sputum	*Kluyvera georgiana*	*Kluyvera georgiana*
27	DRR015591	5.07	54.5	SAMD00012204	-	-	-	-	*Kluyvera georgiana*	*Kluyvera georgiana*
28	WCH1410	4.99	54.8	SAMN04482606	China: Chengdu	environment	2015	hospital sewage	*Kluyvera ascorbata*	*Kluyvera georgiana*
29	DRR015587	4.73	52.6	SAMD00004643	-	-	-	-	*Kluyvera intermedia*	*Kluyvera intermedia*
30	DRR015712	4.66	52.6	SAMD00011660	-	-	-	-	*Kluyvera intermedia*	*Kluyvera intermedia*
31	HR2	4.94	52.3	SAMN13135960	China:Guangzhou	environment	2017	flower	*Kluyvera intermedia*	*Kluyvera intermedia*
32	N2-1	4.94	52.3	SAMN13135738	China:Guangzhou	environment	2017	flower shop	*Kluyvera intermedia*	*Kluyvera intermedia*
33	NBRC 102594	4.64	52.6	SAMD00046734	France	environment	2006	Surface water	*Kluyvera intermedia*	*Kluyvera intermedia*
34	NCTC12125	4.74	52.6	SAMEA2612496	-	-	-	-	*Kluyvera intermedia*	*Kluyvera intermedia*
35	VRES0055-sc-2201594	5.15	52.0	SAMEA3181512	-	-	-	-	*Kluyvera intermedia*	*Kluyvera intermedia*
36	090646	5.48	54.5	SAMN14563383	China: Chengdu, Sichuan	environment	2019	hospital sewage	*Kluyvera sichuanensis*	*Kluyvera sichuanensis*
37	13608	5.03	54.9	SAMN16845436	USA: Lexington, KY	Homo sapiens	2003	sepsis	*Kluyvera sichuanensis*	*Kluyvera sichuanensis*
38	CHPC 1.251*	4.85	54.9	-	China: Beijing	Homo sapiens	2007	faeces	-	*Kluyvera excreta*

39	CHPC 1.254*	5.07	54.8	-	China: Beijing	Homo sapiens	2007	faeces	-	*Kluyvera sichuanensis*
40	CHPC 1.2972*	4.92	55.0	-	China: Beijing	environment	2016	egg surface	-	*Kluyvera chilikensis*
41	Colony413	2.37	55.1	SAMN17914377	Thailand	-	-	food	*Kluyvera ascorbata*	*Kluyvera* sp.
42	L2	4.74	53.4	SAMN03008042	Malaysia	Food	2013	lettuce	*Kluyvera cryocrescens*	*Kluyvera* genomosp. 1
43	CHPC 1.982*	4.96	55.5		China: Beijing	Homo sapiens	2008	faeces	-	*Kluyvera* genomosp. 2
44	KA1	5.28	55.2	SAMN14640326	Spain	Homo sapiens	2014	Human gut	*Kluyvera ascorbata*	*Kluyvera* genomosp. 2
45	KA2	5.26	55.2	SAMN08578632	Spain	Homo sapiens	2014	Rectal colonization	*Kluyvera ascorbata*	*Kluyvera* genomosp. 2
46	KA5	5.30	55.2	SAMN14640327	Spain	Homo sapiens	2014	Human gut	*Kluyvera ascorbata*	*Kluyvera* genomosp. 2
47	MGYG-HGUT-02491	5.26	55.2	SAMEA5851996	Spain	Homo sapiens	-	human gut	*Kluyvera ascorbata*	*Kluyvera* genomosp. 2
48	R10	5.26	55.2	SAMN14640329	Spain	Homo sapiens	2014	Human gut	*Kluyvera ascorbata*	*Kluyvera* genomosp. 2
49	WP4-W19-ESBL-05	5.16	55.1	SAMD00194492	Japan:Tokyo	environment	2019	wastewater	*Kluyvera georgiana*	*Kluyvera* genomosp. 2
50	WP4-W19-ESBL-10	5.44	54.8	SAMD00194497	Japan:Tokyo	environment	2019	wastewater	*Kluyvera georgiana*	*Kluyvera* genomosp. 2
51	WP8-W19-ESBL-13	4.95	55.1	SAMD00194654	Japan:Tokyo	environment	2019	wastewater	*Kluyvera georgiana*	*Kluyvera* genomosp. 2
52	WP8-W19-ESBL-15	4.94	55.3	SAMD00194656	Japan:Tokyo	environment	2019	wastewater	*Kluyvera georgiana*	*Kluyvera* genomosp. 2
53	CRP	5.16	54.8	SAMN21013960	Hong Kong: Ocean Park	environment	2018	Ailurus fulgens faeces	*Kluyvera sp.*	*Kluyvera* genomosp. 3
54	ERR3712946	5.08	54.7	SAMEA6368553	Netherlands	-	-	-	*Kluyvera georgiana*	*Kluyvera* genomosp. 3

55	PO2S7	5.00	54.9	SAMN13951572	India:Manipur	environment	2018	Oryza sativa	*Kluyvera genomosp. 3*	*Kluyvera* genomosp. 3
56	YDC799	5.00	55.0	SAMN07267279	USA: Pittsburgh, PA	Homo sapiens	2017	-	*Kluyvera georgiana*	*Kluyvera* genomosp. 3
57	D51-sc-1712206	4.80	52.9	SAMEA2393042	-	-	-	-	*Kluyvera cryocrescens*	*Kluyvera* genomosp. 4
58	e02842a0-063f-11e6-bb80-3c4a9275d6c8	4.76	52.8	SAMEA104428612	-	Homo sapiens	-	gut	*Kluyvera cryocrescens*	*Kluyvera* genomosp. 4
59	169	5.20	53.3	SAMN16845438	Argentina: Buenos Aires	Homo sapiens	2003	Human, lower urinary tract infection	*Kluyvera sp.*	*Kluyvera* genomosp. 5
60	RHBSTW-00307	5.23	53.4	SAMN16088452	United Kingdom	environment	2017	wastewater influent	*Kluyvera cryocrescens*	*Kluyvera* genomosp. 5

Note: Strains labeled in dark blue are misidentified.

Table S2 Primer sequence information used in this study

Strain	Primer	Enzyme digestion sites	Sequence (5′–3′)	Target sequence (bp)
CHPC 1.251	KLUS$_{CHPC\ 1.251}$-F	*XbaI*	GCTCTAGAATGATGACCCTGGGCGTTCG	873
	KLUS$_{CHPC\ 1.251}$-R	*HindIII*	CCCAAGCTTCTAAAGCCCTTCGGTAACGATTTTTGC	
CHPC 1.2972	KLUS$_{CHPC\ 1.2972}$-F	*XbaI*	GCTCTAGAATGATGACCCTGGGCGTTCG	873
	KLUS$_{CHPC\ 1.2972}$-R	*HindIII*	CCCAAGCTTCTAAAGCCCTTCGGTAACGATTTTGGC	
CHPC 1.254	KLUS$_{CHPC\ 1.254}$-F	*XbaI*	GCTCTAGAATGATGACCCTGGGCGTTCG	873
	KLUS$_{CHPC\ 1.254}$-R	*HindIII*	CCCAAGCTTCTAAAGCCCTTGGGTAACGATTTTTGC	

Table S3 Phenotypic characteristics of *K. sichuanensis* 090646^T, *K. excreta* CHPC 1.251^T and *K. chilikensis* CHPC 1.2972^T

Characteristic	*K. sichuanensis* 090646^T	*K. excreta* CHPC 1.251^T	*K. chilikensis* CHPC 1.2972^T
Motility	+	+	+
Indole production*	−	+	+
Voges–Proskauer reaction	−	−	−
Citrate utilization	+	+	+
H_2S production	−	−	−
Malonate utilization	+	+	+
NO3->NO2	+	+	+
ONPG test	−	−	−
Oxidase	−	−	−
Catalase	−	−	−
Lipase	−	−	−
Sorbitol fermentation*	+	−	−
β-D-Glucosidase (ESC)	+	+	+
β-galactosidase (PNPG)	+	+	+
Urease	−	−	−
Lysine decarboxylase	+	+	+
Arginine dihydrolase	−	−	−
Ornithine decarboxylase	+	+	+
Gelatinase (GEL)	−	−	−
**Acid production from:**			
D-glucose	+	+	+
Sucrose*	−	+	+
Dulcitol	−	−	−
D-sorbitol	+	+	+
Amygdalin	+	+	+
Glycerol	−	−	−
D-mannose	−	−	−

D-galactose	+	+	+
Esculin	+	+	+
methyl α-D-glucopyranoside	+	+	+
**Assimilation of:**			
Glucose	+	+	+
Arabinose (ARA)	+	+	+
D-Mannose (MNE)	+	+	+
D-Mannitol (MAN)	+	+	+
N-Acetyl-D-glucosamine (NAG)	+	+	+
Maltose (MAL)	+	+	+
Gluconate (GNT)	+	+	+
Mannitol	+	+	+
α-Glucosidase*	-	-	+

Note: The biochemical reactions marked with "*" are the differential biochemical indicators of the three species.

Table S4 The results of antibiotic susceptibility testing.

	AMI	AMP	A/S2	AZM	AZT	CTX	FOX	CAZ	CAP	CIP	CT	ETP	IPM	MEM	NA	NIT	S	TET	TGC	SXT	CAZ-NXL
CHPC1.251	<=4	8	2/1	16	<=0.25	<=0.25	<=2	<=0.25	<=4	<=0.01563	0.5	<=0.25	<=0.25	<=0.125	<=4	<=32	8	2	<=0.25	<=0.5/9.5	<=0.25/4
CHPC1.254	<=4	8	2/1	32	<=0.25	<=0.25	<=2	<=0.25	<=4	<=0.01563	0.5	<=0.25	<=0.25	<=0.125	<=4	<=32	8	2	<=0.25	<=0.5/9.5	<=0.25/4
CHPC1.2972	<=4	16	2/1	32	<=0.25	<=0.25	<=2	<=0.25	<=4	<=0.01563	0.5	<=0.25	<=0.25	<=0.125	<=4	<=32	8	2	<=0.25	<=0.5/9.5	<=0.25/4
CHPC1.982	<=4	<=2	2/1	16	<=0.25	<=0.25	<=2	<=0.25	<=4	<=0.01563	0.5	<=0.25	0.5	<=0.125	<=4	<=32	<=4	<=1	<=0.25	<=0.5/9.5	<=0.25/4

Note: Amikacin: AMI; Ampicillin: AMP; Ampicillin-Sulbactam: A/S2; Azithromycin: AZM; Aztreonam: AZT; Cefotaxime: CTX; Cefoxitin: FOX; Ceftazidime: CAZ; Chloramphenicol: CAP; Ciprofloxacin: CIP; Colistin: CT; Ertapenem: ETP; Imipenem: IPM; Meropenem: MEM; Nalidixic Acid: NA; Nitrofurantoin: NIT; Streptomycin: S; Tetracycline: TET; Tigecycline: TGC; Trimethoprim-Sulfamethoxazole: SXT; ceftazidime-avibactam: CAZ-NXL
